# Dr. Wm. Lucius Hunter

**Published:** 1915-06

**Authors:** 


**Dr. Wm. Lucius Hunter,** Buffalo 1867, died at his home in Brooklyn, April 22, aged 65, of nephritis. He was formerly a clergyman of the African M. E. Church.

